# Warm-Up With Added Respiratory Dead Space Volume Mask Improves the Performance of the Cycling Sprint Interval Exercise: Cross-Over Study

**DOI:** 10.3389/fphys.2022.812221

**Published:** 2022-03-15

**Authors:** Natalia Danek, Kamil Michalik, Marek Zatoń

**Affiliations:** ^1^Department of Physiology and Biochemistry, Faculty of Physical Education and Sport, Wrocław University of Sport and Health Sciences, Wrocław, Poland; ^2^Department of Human Motor Skills, Faculty of Physical Education and Sport, Wrocław University of Sport and Health Sciences, Wrocław, Poland

**Keywords:** warm-up, added respiratory dead space volume mask, hypercapnia, sprint interval exercise, performance, respiratory muscles

## Abstract

Special breathing exercises performed during warm-up lead to hypercapnia and stimulation of mechanisms leading to increased exercise performance, but the effect of a device that increases the respiratory dead space volume (ARDSv) during warm-up has not been studied. The purpose of this study was to investigate the effect of 10 min warm-up with ARDSv on performance, physiological and biochemical responses during sprint interval cycling exercise (SIE). During four laboratory visits at least 72 h apart, they completed: (1) an incremental exercise test (IET) on a cycloergometer, (2) a familiarization session, and cross-over SIE sessions conducted in random order on visits (3) and (4). During one of them, 1200 mL of ARDSv was used for breathing over a 10-min warm-up. SIE consisted of 6 × 10-s all-out bouts with 4-min active recovery. Work capacity, cardiopulmonary parameters, body temperature, respiratory muscle strength, blood acid-base balance, lactate concentration, and rating of perceived exertion (RPE) were analyzed. After warm-up with ARDSv, P_*ET*_CO_2_ was 45.0 ± 3.7 vs. 41.6 ± 2.5 (mm Hg) (*p* < 0.001). Body temperature was 0.6 (°C) higher after this form of warm-up (*p* < 0.05), bicarbonate concentration increased by 1.8 (mmol⋅L^–1^) (*p* < 0.01). As a result, work performed was 2.9% greater (*p* < 0.01) compared to the control condition. Respiratory muscle strength did not decreased. Warming up with added respiratory dead space volume mask prior to cycling SIE produces an ergogenic effect by increasing body temperature and buffering capacity.

## Introduction

High-Intensity Interval Training (HIIT) is an alternative form of improving physical performance in a shorter period of time than moderate intensity exercise (Metcalfe et al., 2012; Gillen et al., 2014).

In general, interval training involved a few repeated efforts of high intensity interspersed with active or passive recovery periods (Billat, 2001). HIIT programs are categorized into long- or short-duration programs of varying intensities, according to Buchheit and Laursen (2013). One of the most popular types of HIIT short duration is sprint interval training (SIT), which involves performing work at maximal intensity (generating the highest possible power, known as “all-out”) (Gibala and Hawley, 2017). A single session of SIT (sprint interval exercise—SIE) typically consists of two to six bouts lasting between 6 and 30 s, with the recovery of 2–4 min duration and a total duration of SIE of typically 10–30 min (Danek et al., 2020b; Hall et al., 2020). Given that these efforts are characterized by short repetitive attacks of maximal intensity, it is advisable to use a warm-up to prevent the occurrence of injuries, ensuring that the expected performance is achieved during the main part of the exercise session (Woods et al., 2007). A particularly important aspect is the preparation of an appropriate warm-up before the acute sprint interval exercise (Frikha et al., 2016).

Both passive and active warm-up procedures aims to optimize body temperature, metabolic, neurological, and psychological responses. One of the warm-up aims is to increase blood flow through working muscles through e.g., vasodilation (Neiva et al., 2014), in order to optimize metabolic responses, thereby improving resting intramuscular concentrations of the adenine nucleotidesduring (adenosine triphosphate, adenosine diphosphate, adenosine monophosphate) acute exercise (Febbraio et al., 1996), and for faster deoxygenation of hemoglobin (Bishop, 2003). The warming process increases baseline oxygen uptake (VO_2_) (Burnley et al., 2011) and improves nerve conduction velocity (Pearce et al., 2012). All of the effects described are mainly attributed to increases in body and muscle temperature (Bishop, 2003). However, it is believed that the aforementioned mechanisms should not cause excessive fatigue, but rather prepare the body for the best possible exercise performance.

Various treatments (provoked adaptive changes) are used to improve potential exercise effects. These include regulating the composition of inhaled air using various types of breathing manipulations. Woorons et al. (2007) used an apneas cycle during submaximal exercise to induce hypoxemia, leading to delayed metabolic acidosis and increased exercise performance. In addition, exercise in hypoxia is thought to produce compensatory vasodilation with an induced nitric oxide-dependent increase in muscle blood flow (Casey and Joyner, 2012). Following this line of thought, Lemaître et al. (2010) proposed the apnea cycle as a new method/form of warm-up (preparation for exercise) inducing tolerable hypoxia. This response occurs in conjunction with hypercapnia, increased respiratory acidosis, bradycardia, and splenic contraction due to hypoxemia during the initial apneic phase, among other things (Baković et al., 2003). This leads to an increase in the number of circulating erythrocytes, suggesting a potential method to rapidly increase the body’s oxygen transport capacity. In contrast, Bahenský et al. (2020) tested breathing exercises based on Wim Hof method, which is a combination of deep breathing and breath-holding, increasing the concentration of carbon dioxide in arterial blood and inducing hypercapnia. Induction of hypercapnia prior to exercise is thought to elicit a sympathetic response leading to increased tidal volume (VT) and increased blood flow to skeletal muscle in a manner that may improve exercise performance. In turn, increased partial pressure of carbon dioxide (pCO_2_) in the body increases the concentration of bicarbonate in the blood (Woorons et al., 2007, 2010). This may affect buffering capacity and may be beneficial for pH regulation and stimulating anaerobic metabolic efficiency, especially during intense exercise, e.g., repeated sprints (Trincat et al., 2017).

During sprint interval exercise session Danek et al. (2020a) did not use affected respiratory modulation but applied a special device, added respiratory dead space volume 1200 mL, to provoke hypercapnia. This led to an ergogenic effect in the form of a higher bicarbonate concentration resulting in a higher average power (total work) during the SIE, at the same time accompanied by increased physiological responses (e.g., higher VO_2_peak, lower pH) with a lower rating of perceived exertion (RPE) compared to standard conditions. Furthermore, breathing through such a device causes some of the exhaled and heated air to remain in that space, mix with the inhaled air, and increase the percentage of CO_2_ in that air, which may increase its temperature and body temperature. However, these physiological responses have not been studied. Koppers et al. (2006) using “tube breathing” (external dead space ventilation) and controlled breathing (modulating the breathing rhythm—15 breaths per minute in a 1:2 inspiratory:expiratory rhythm) to improve respiratory muscle function, they found that this also led to hypercapnia (pCO_2_ ≤ 50 mm Hg) in most participants, which is well tolerated by healthy individuals. On the other hand, in our study (Danek et al., 2020a), the SIE session with ARDSv did not put additional stress on the respiratory muscles, as no decrease in their strength was noted. Therefore, it seems interesting to study acid-base imbalance and respiratory muscle strength e.g., during warm-up on physical parameters during sprint interval exercise.

To date, the effect of warming up with ARDSv before sprint interval exercise has not been verified; therefore, the purpose of this study was to compare acute physiological, biochemical, and perceptual responses during a SIE session preceded by a 10-min warm-up with and without a device increasing the respiratory dead space volume by 1200 mL. We hypothesized that using 1200 mL of ARDS_*V*_ during the warm-up would provoke hypercapnic acidosis, which in turn would initiate disrupt the acid–base balance, leading to increased bicarbonate concentrations and blood buffering capacity. Also, inhaling heated air would induce an increase in body temperature. On the other hand, increased respiratory activity (VT) will be reflected in a higher VO_2_, but the increased ventilatory work will not result in respiratory muscle fatigue. These responses would induced ergogenic effects and increase performance during the cycling sprint interval exercise.

## Materials and Methods

### Participants

The study involved 10 healthy (aged 21–32 years), physically active men who volunteered to participate. Each declared a minimum of 5 h of physical exercise (college sports, gym, volleyball, soccer, running) per week. None played sports at the professional level and were classified as being at risk for respiratory, cardiovascular, and metabolic diseases. Tobacco smokers were not among them. All of them were familiarized with the study procedure and gave written informed consent to participate. The study was approved by the University Research Ethics Committee (1/2019) and was conducted in accordance with the Declaration of Helsinki (EN ISO 9001 certified: 2001). Detailed characteristics of the participants are shown in Table 1.

**TABLE 1:** Participants’ characteristics ($\bar{x}\pm \mathrm{SD}$).

Variables	($\bar{\text{x}}\pm \text{SD}$)
Age (Years)	24.6 ± 4.1
Body height (cm)	180.4 ± 8.1
Body mass (kg)	77.3 ± 11.4
Physical activity (h per week)	7.7 ± 1.5
Systolic blood pressure (mm Hg)	125 ± 10
Diastolic blood pressure (mm Hg)	70 ± 9
MAP (W)	337.4 ± 43.0
VO_2_max (mL⋅kg^–1^⋅min^–1^)	52.5 ± 8.6
VEmax (L⋅min^–1^)	149.7 ± 23.0
VTmax (L)	3.4 ± 0.5
RFmax (breath⋅min^–1^)	51.7 ± 8.0
HRmax (beats⋅min^–1^)	192 ± 6
FVC (L)	6.9 ± 1.0
FEV_1_ (L)	5.0 ± 0.9
FEV_1_⋅FVC^–1^ (%)	73.1 ± 9.9
PIF (L⋅s^–1^)	3.1 ± 1.5
PEF (L⋅s^–1^)	9.0 ± 1.2
RBC (10^6^⋅mm^–3^)	5.1 ± 0.5
HGB (gl⋅dL^–1^)	15.1 ± 0.8
Hct (%)	44.1 ± 3.3

*MAP, maximal aerobic power; VO_2_max, maximal oxygen uptake; VEmax, maximal respiratory minute ventilation; VTmax, maximal tidal volume; Rfmax, maximal respiratory rate; FVC, forced vital capacity; FEV_1_, forced expiratory volume in 1 s; FEV_1_⋅FVC^–1^, Tiffeneau index; PIF, peak inspiratory flow; PEF, peak expiratory flow; RBC, red blood cells; HGB, hemoglobin concentration; Hct, hematocrit.*

### Study Design

The study included four laboratory visits at least 72 h apart, conducting cycle-ergometer exercise sessions over their course. All sessions were conducted by the same researchers and performed in the morning, 2 h after breakfast. During the experiment, participants maintained their current physical activity and were asked to abstain from strenuous exercise, alcohol and caffeine for 24 h before each session in the lab. During the first visit body mass (kg) and height (cm) were measured using a WPT 200 medical balance (RADWAG, Radom, Poland), resting blood pressure was measured using an aneroid sphygmomanometer (Riester, Jungingen, Germany), spirometry test and incremental exercise test (IET) was performed on a cycle-ergometer to determine cardiorespiratory capacity. The second visit included familiarization with a cycling sprint interval exercise (SIE) protocol and breathing with additional respiratory dead space volume mask (ARDSv). At the third and fourth visits, single cross-over SIE sessions were conducted, during which, in random order, warm-ups were performed with (SIE_*ARDSwu*_) and without ARDSv (CON).

### Hematological Parameters

Capillary blood was collected from the fingertip of the hand before the exercise test at rest for determination of blood morphological parameters: hemoglobin concentration (HGB) and hematocrit (Hct) using red blood cells (RBC) an ABX Micros OT.16 (Horiba Medical, Japan).

### Spirometry Test

The spirometric test was performed with a Quark b^2^ ergospirometer (Cosmed, Milan, Italy). It consisted of a maximum-volume inhalation preceded by two to three calm breaths and ending with a forced exhalation of maximum airflow leading to a minimum residual air volume. Peak expiratory flow (PEF), peak inspiratory flow (PIF), forced vital capacity (FVC), and first-second expiratory volume (FEV_1_) were recorded during the respiratory test. Each participant completed three trials. The first trial was a familiarization exercise. The program calculated the Tiffeneau index (FEV_1_⋅FVC^−1^).

### Incremental Exercise Test

The test was performed on an Excalibur Sport cycle-ergometer (Lode BV, The Netherlands) according to the RAMP protocol with a linear increase in load. It started with a load of 0 W, which increased every second by another ∼0.278 W (corresponding to 50 W⋅ 3 min^–1^) (Michalik et al., 2019). The minimum pedaling frequency was 60 revolutions per minute. The test continued until volitional exhaustion.

The participant breathed through a mask, and the expired air was analyzed by a Quark b^2^ device (Cosmed, Milan, Italy). The device was calibrated with atmospheric air and gas mixture of composition: 5% CO_2_, 16% O_2_, and 79% N_2_, before the measurements began. The recording of respiratory parameters was performed breath by breath. Pulmonary ventilation (VE), respiratory rate (Rf), tidal volume (VT), and oxygen uptake (VO_2_) were measured, and the results were averaged every 30 s, and converted to minute values. Heart rate (HR) was measured using a S810 sport-tester (Polar Electro, Kempele, Finland) and recorded with Quark b^2^ analyzer software. VO_2_max was recorded as the highest 30-s mean value with a VO_2_ plateau < 1.35 mL⋅kg^–1^⋅min^–1^ despite increasing load or when a minimum of two criteria were met: (1) volitional exhaustion, (2) predicted HRmax ≥ 95% (220—age), 3) respiratory rate ≥ 1.10. Maximal aerobic power (MAP) was determined as power during attainment maximal oxygen uptake.

Capillary blood was drawn from the fingertip of the hand into heparinized capillaries at rest and at the third minute after the test for determination of lactate ([La^–^]) on a photometer (LP 400 Dr Lange, Germany).

### Cycling Sprint Interval Exercise Sessions

**FIGURE 1:**
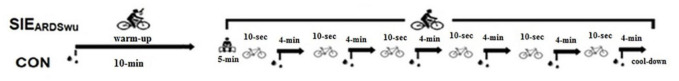
Cycling sprint interval exercise protocols.

Both sessions were performed on a cycle ergometer (Ergomedic Monark 894, Vansbro, Sweden) according to the protocol described by Danek et al. (2020a) and shown in Figure 1. Prior to the SIE, a 10-min warm-up was applied at an intensity of 60% of the maximal aerobic power obtained in the IET, during which two 5-s “all-out” accelerations were performed with a load of 7.5% of body weight, in the third and sixth minutes. The warm-up was followed by 5 min of rest in a sitting position. In the main part, participants performed six 10-s bouts with an individual load of 7.5% of body weight and an active 4-min break. Intervals between bouts and in cool-down were conducted with a load of 50 W and a revolution frequency of 50 rpm. The participants were encouraged by loud shouts to perform as much effort as possible during the bouts. The schematic is shown in Figure 1.

Using a Quark b^2^ (Cosmed, Milan, Italy), respiratory gases were measured analogously to the IET. In addition, the following were measured: oxygen percentage in inspired air (FiO_2_), carbon dioxide percentage in inspired air (FiCO_2_), end-expiratory partial pressure of oxygen (P_*ET*_O_2_), and end-expiratory partial pressure of carbon dioxide (P_*ET*_CO_2_). Heart rate (HR) was recorded throughout the session using a S810 sports-tester (Polar Electro, Kempele, Finland). The results were averaged over 30-s intervals and converted to minutes. The measurements of ergospirometry was carried out according to Danek et al. (2020a).

Capillary blood was collected from the fingertip of the hand into heparinized capillaries at the third minute after the warm-up and after each sprint to determine: (a) acid-base balance: pH, partial pressure of carbon dioxide (pCO_2_), current bicarbonate concentration [HCO_3_^–^] and blood oxygen saturation SaO_2_ using a RapidLab 348 analyzer (Bayer, Germany), (b) lactate concentration ([La^–^]) on a photometer (LP 400 Dr Lange, Germany), regarding to Goodwin et al. (2007) peak lactate can be determined in third minutes after exercise.

Body temperature (T) was measured on the frontal tubers according to the study procedure of Hebisz et al. (2019) at rest, after warm-up, and after the sixth bout using a non-contact thermometer (VisioFocus Smart, Italy). The device has been validated and shown to provide reliable measurements of body temperature on the skin (Morán-Navarro et al., 2019).

### Warm-Up With Added Respiratory Dead Space Volume Mask

During one session, an added respiratory dead space volume mask of 1200 mL (SIE_*ARDSwu*_) was used in the warm-up. It was formed by a single-valve Ambu-type mask, and an attached ribbed tube with a 2.5 cm diameter. The dead space volume mask thus created was identical for each participant and measured by filling the pipe with water, and then transferring the volume to a measuring cylinder according to Szczepan et al. (2020). The ARDSv was put on 2 min before the start of the warm-up and taken off 2 min before the start of the first bout. The total time with ARDSv in place was 15 min. The second session took place under standard conditions without ARDSv (CON).

### Performance

The results obtained during both SIEs were analyzed in terms of peak power output (PPO), mean power output (MPO), and total work (Wtot). They were calculated using MCE 2.0 software (MCE, Wrocław, Poland) for six replicates. Fatigue levels were estimated by calculating the power loss index (FI) according to the formula:


F⁢I=(100×(t⁢o⁢t⁢a⁢l⁢s⁢p⁢r⁢i⁢n⁢t⁢M⁢P⁢O÷i⁢d⁢e⁢a⁢l⁢s⁢p⁢r⁢i⁢n⁢t⁢M⁢P⁢O))-100

where total sprint MPO = sum of sprint MPO from all sprints; ideal sprint MPO = number of sprints (6) × the highest sprint MPO.

This formula was found to be the most valid and reliable method for assessing fatigue in multiple sprint tests (Glaister et al., 2008).

### Respiratory Muscle Strength Variable Measurements

Inspiratory muscle strength (PImax)—maximal inspiratory pressure (cm H_2_O) and expiratory muscle strength (PEmax)—maximal expiratory pressure (cm H_2_O) were measured using a Micro RPM pressure monitor (CareFusion, San Diego, CA, United States). To assess PImax, the participant in a standing position performed maximal inhalation starting from the maximal expiratory level. Then, to assess PEmax, the participant exhaled, starting at the maximal inspiratory level. A nose clip was used in both cases to prevent airflow through this route. PImax and PEmax tests were performed at rest before warm-up and at 10 min after SIE. Each participant performed two trials of maximal inspiration and maximal expiration, and the higher values were selected for further analysis.

### Borg Ratings of Perceived Exertion Scale

The Ratings of Perceived Exertion (RPE) is a tool for subjectively assessing the perception of exercise intensity, or fatigue level (Borg, 1982). This scale allows you to relate the degree of fatigue during training to the fatigue experienced during daily activities. In general, a score > 18 indicates that a maximal effort was performed, and values > 15–16 indicate that the anaerobic threshold was exceeded. The scores on this scale refer to heart contraction rate. The principle of the scale is to divide the predicted HR for a given effort by 10, hence an effort causing a heart rate increase to 190 beats⋅min^–1^ receives 19 points, and a complete rest in which the HR oscillates between 60 and 70 beats⋅min^–1^–6–7 points. RPE was measured immediately after the warm-up and after each repetition of the SIE. RPE was determined according to others research using sprint interval exercises (Flores et al., 2018).

### Statistical Analysis

Mean values of cardiorespiratory parameters in both SIE sessions were calculated for 10 min of warm-up and for 25 min of the main part (1 min of activity, 20 min in active recovery, and 4 min of passive recovery). Mean values of pH, [La^–^], [HCO_3_^–^], SaO_2_, RPE, and T were calculated from measurements taken after warm-up. Additionally, RPE and T were measured after the sixth bouts.

Statistica 13.3 software (StatSoft Inc., Tulsa, OK, United States) was used to statistically process the data. Results are presented as the arithmetic mean ± standard deviation ($\bar{\text{x}}$ ± SD). Shapiro-Wilk Test was used to assess the normality of the distribution of the studied characteristics, whereas the homogeneity of variance was assessed by Levene’s test. Student’s *t*-test for dependent samples was used to assess differences in selected variables between SIE protocols. Effect size (ES) Cohen’s d was calculated in order to show practical effect, using the following criteria: 0.1—trivial, 0.2—small, 0.5—medium, 0.8—large (Cohen, 1988). A two-factor (protocol × bout) analysis of variance with repeated measures (RM-ANOVA) was used to compare PPO, pH, and [La^–^] during SIE, as well as changes in respiratory muscle strength. A Bonferroni *post-hoc* test was performed when a significant F ratio value was obtained. For significant differences, the effect size was calculated as eta-square (η^2^) (small = 0.01, moderate = 0.13, high = 0.26). The *p* < 0.05 level was considered statistically significant.

## Results

Baseline pH (*p* = 0.57, *t* = 0.58), pCO_2_ (*p* = 0.82, *t* = 0.23), [HCO_3_^–^] (*p* = 0.90, *t* = 0.12), T (*p* = 0.79, *t* = −0.29) were not statistically significantly different between conditions.

Table 2 shows a comparison of the mean values of cardiorespiratory parameters, lactate concentration and blood gas tests, body temperature, as well as RPE after the warm-up.

**TABLE 2:** Comparison of mean physiological responses between warm-up in the both SIE protocols.

Variables	CON	SIE_*ARDSwu*_	*p*-value	*t*-test	ES
Fi_*O2*_ (%)	20.8 ± 0.05	20.6 ± 0.08*	<0.001	11.3	3.95
Fi_*CO2*_ (%)	0.06 ± 0.03	0.20 ± 0.07*	<0.001	6.3	2.51
P_*ET*_CO_2_ (mm Hg)	41.6 ± 2.5	45.0 ± 3.7*	<0.001	5.18	1.36
P_*ET*_O_2_ (mm Hg)	99.8 ± 3.4	93.5 ± 4.1*	<0.001	4.93	1.66
Rf (breath⋅min^–1^)	24.4 ± 2.1	24.8 ± 3.4	0.64	0.48	0.11
VT (L)	2.4 ± 0.3	2.7 ± 0.5*	<0.001	4.47	0.87
VE (L⋅min*^–^*^1^)	57.8 ± 4.4	66.3 ± 8.2*	<0.001	4.11	1.29
VO_2_ (mL⋅kg*^–^*^1^⋅min^–1^)	30.4 ± 2.8	39.6 ± 6.9*	<0.001	6.31	1.77
HR (beats⋅min^–1^)	139 ± 15	140 ± 12	0.76	0.31	0.08
RPE (6–20)	9 ± 3	10 ± 2	0.53	0.65	0.23
[La^–^] (mmol⋅L^–1^)	4.39 ± 2.1	3.5 ± 1.2	0.11	1.77	0.55
pH	7.40 ± 0.03	7.37 ± 0.03*	<0.01	3.06	0.87
pCO_2_ (mm Hg)	38.2 ± 3.4	44.0 ± 2.4*	<0.001	5.99	1.96
[HCO_3_*^–^*] (mmol⋅L^–1^)	22.9 ± 2.2	24.7 ± 1.6*	<0.01	3.03	0.92
SaO_2_ (%)	96.1 ± 1.0	95.2 ± 0.9*	<0.05	2.38	0.95
T (°C)	36.4 ± 0.4	37.0 ± 0.7*	<0.05	2.44	1.14

*Parameters are presented as arithmetic means and standard deviation ($\bar{x}\,S\,D$). Fi_O2_, percentage of oxygen in the inspiratory air_;_ Fi_CO2_, percentage of carbon dioxide in the inspiratory air; P_ET_CO_2_, end-expiratory partial pressure of carbon dioxide_;_ P_ET_O_2_, end-expiratory partial pressure of oxygen; Rf, respiratory rate; VT, tidal volume; VE, respiratory minute ventilation; VO_2_, oxygen intake; HR, heart rate; RPE, Rated Perceived Exertion; [La^–^], lactate concentration; pCO_2_, carbon dioxide partial pressure in capillary blood; [HCO_3_^–^], current bicarbonate concentration; SaO_2_, blood saturation, oxygen saturation of hemoglobin; T, body temperature.*

*Statistically significant difference (p < 0.05) between CON and SIE_ARDS_wu.

**FIGURE 2:**
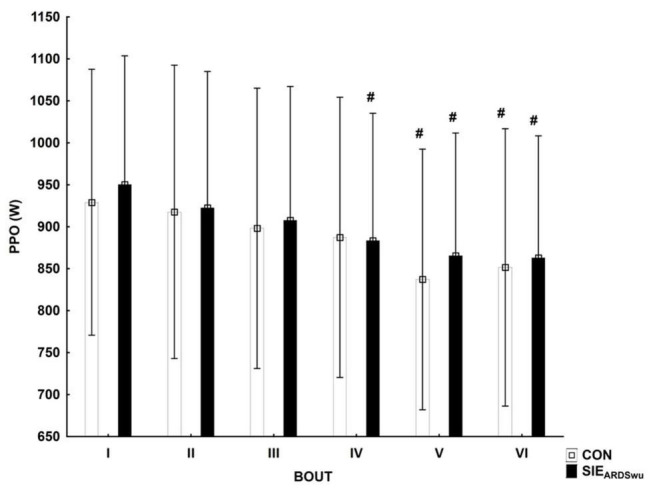
Peak power output (PPO) in consecutive bouts during a SIE session. ^#^Statistically significant difference relative to the first bout (*p* < 0.05).

Figure 2 shows the PPO during six bouts in both conditions tested. A main effect was found for BOUT [*F*_(5, 90)_ = 17.571, *p* < 0.001, η^2^ = 0.49]. The *post-hoc* analysis showed a statistically significant reduction in peak power relative to first bout in CON during V (*p* < 0.001) and VI (*p* < 0.001), as well as in SIE_*ARDSwu*_ in IV (*p* < 0.05), V, and VI (*p* < 0.001 in both). No PROTOCOL × BOUT interaction was diagnosed [*F*_(5, 90)_ = 0.57, *p* = 0.72].

**FIGURE 3:**
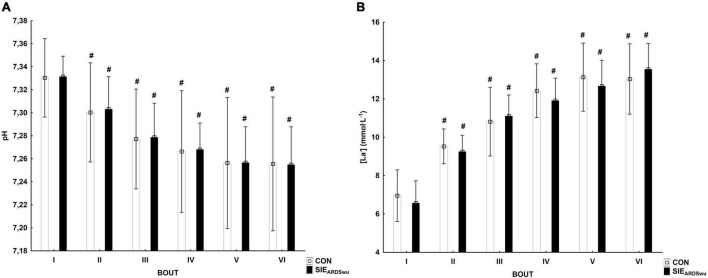
Changes in pH **(A)** and lactate concentration (mmol⋅L^−1^) **(B)** in consecutive bouts during SIE sessions. ^#^Statistically significant difference relative to the first bout (*p* < 0.05).

There was a main effect for BOUT [*F*_(5, 90)_ = 41.0, *p* < 0.001, η^2^ = 0.69] for blood pH, which decreased statistically significantly relative to the first bout during CON in III (*p* < 0.05), IV (*p* < 0.01), V (*p* < 0.001), and VI bout (*p* < 0.001). SIE_*ARDSwu*_ also showed significantly lower blood pH starting from III to VI bout (*p* < 0.001 for all) compared to the first one (Figure 3A). There were no differences between the tested warm-ups in any bout. Changes in blood lactate concentration are shown in Figure 3B, where the main effect for BOUT was recorded [*F*_(5, 90)_ = 125.69, *p* < 0.001, η^2^ = 0.87]. Lactate concentration increased starting from the second bout in both groups, and was statistically significantly higher in subsequent bouts (*p* < 0.001 for all).

Mean power was statistically significantly higher in SIE_*ARDSwu*_ (771.7 ± 147.8 W) compared to CON (749.6 ± 138.7 W) (*p* = 0.01, *t* = 3.04, ES = 0.24). The amount of work performed was statistically significantly different between protocols (*p* = 0.01, *t* = 3.05, ES = 0.24), 46.3 ± 8.9 and 45.0 ± 8.3 kJ, in SIE_*ARDSwu*_ and CON, respectively. There was no statistically significant difference between FIs (*p* = 0.68, *t* = 0.42). RPEmean (*p* = 0.12, *t* = 1.72), RPEpeak (*p* = 0.89, *t* = 31.79), [La^–^] mean (*p* = 0.80, *t* = 0.25) and [La^–^] peak (*p* = 0.93, *t* = 0.09) recorded in the main part were not statistically significantly different between sprint interval exercise sessions.

**FIGURE 4:**
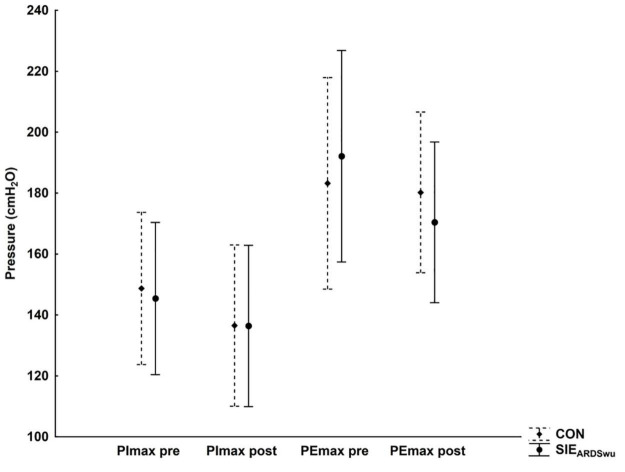
Changes in inspiratory (PImax) and expiratory (PEmax) muscle strength at rest and after exercise under CON and SIE_*ARDSwu*_ conditions.

Respiratory muscle strength (PImax and PEmax) was not statistically significantly different between conditions both before, and after the entire intervention (Figure 4).

## Discussion

The purpose of this study was to determine the effects of a hypercapnic warm-up prior to sprint interval exercise on changes in oxygen uptake, amount of work performed and power generated, and RPE during six 10-s bouts on a cycle-ergometer. Despite previous analysis of the effects of ARDSv, e.g., on cardiovascular and respiratory activity during sprint interval exercise (Danek et al., 2020a), this is the first study to verify the use of this device and provoked conditions during warm-up. Analysis of the changes in the values of the studied characteristics showed that the use of ARDSv during a 10-min warm-up leads to higher values of VO_2_, VE, VT. In contrast, it does not affect the weakening of respiratory muscle strength. The use of ARDSv also leads to lower blood pH compared to CON, but this does not contribute to a statistically significant reduction [La^–^]. It does not induce an increase in RPE, either immediately after the warm-up or after the main part of the SIE. Additionally, performing a warm-up under significantly elevated pCO_2_ compared to CON leads to statistically significantly higher bicarbonate concentrations and to the maintenance of higher body temperature. All of the foregoing changes induced by the hypercapnic warm-up resulted in a statistically significantly higher mean power and amount of work performed, with a lower mean oxygen uptake (Table 3) during the main part of the SIE (six 10-s “all-out” bouts), proving the ergogenic effect.

**TABLE 3:** Comparison of mean and peak physiological responses between both SIE protocols (25 min).

Variables	CON	SIE_*ARDSwu*_	*p*-value	*t*-test	ES
P_*ET*_CO_2_ (mm Hg)	32.7 ± 3.5	33.1 ± 3.2	0.57	0.58	0.11
Rf (breath⋅min^–1^)	30.9 ± 3.5	30.6 ± 3.4	0.71	0.38	0.08
VT (L)	2.5 ± 0.4	2.6 ± 0.4	0.24	1.26	0.15
VEmean (L⋅min^–1^)	77.3 ± 12.8	77.1 ± 10.8	0.72	0.37	0.07
VEpeak (L⋅min^–1^)	118.6 ± 20.8	114.6 ± 20.9	0.20	1.37	0.20
VO_2_ mean (mL⋅kg^–1^⋅min^–1^)	28.0 ± 2.4	26.8 ± 2.5*	<0.05	2.4	0.51
VO_2_ peak (mL⋅kg^–1^⋅min^–1^)	43.3 ± 5.1	42.4 ± 6.5	0.16	1.55	0.16
HR mean (beats⋅min^–1^)	152 ± 13	152 ± 12	0.94	0.08	0.02
HR peak (beats⋅min^–1^)	177 ± 8	176 ± 7	0.59	0.56	0.14
T (°C)	36.0 ± 0.6	36.0 ± 0.3	0.82	0.23	0.10

Parameters are presented as arithmetic means and standard deviation ($\bar{x}\,S\,D$). P_ET_CO_2_, end-expiratory partial pressure of carbon dioxide_;_ Rf, respiratory rate; VT, tidal volume; VE, respiratory minute volume; VO_2_, oxygen uptake; HR, heart rate; T, body temperature. *Statistically significant difference (p < 0.05) between CON and SIE_ARDSwu_.

When analyzing the effect of breathing with 1200 mL of ARDSv during a 10 min warm-up, we found a significant increase in VO_2_, FiCO_2_, and pCO_2_ and a decrease in FiO_2_ and P_*ET*_O_2_ compared to control conditions. Nevertheless, despite a significant reduction in hemoglobin oxygen saturation (0.9%), we did not observe hypoxemia or hypoxia (SaO_2_ > 95%). This phenomenon was observed by Woorons et al. (2010) during hypoventilation. Modulation of the respiratory rate led to respiratory acidosis and a consequent rightward shift of the oxygen-hemoglobin dissociation curve (ODC), which emphasizes the reduction of SaO_2_. In contrast, the use of airflow restriction mask (ARM) in the randomized study by Romero-Arenas et al. (2021) did not significantly affect SaO_2_ in muscles, but increased oxygen delivery to frontal cortex, as measured by near-infrared spectroscopy. The authors indirectly attributed this phenomenon to a mask-induced hypercapnia state, but they did not measure blood pCO_2_. Barbieri et al. (2020), when using ARM during 20 min of continuous exercise at an intensity of 60%VO_2_max, obtained similar results to ours, but despite the demonstrated significant differences in pCO_2_, the values achieved were 15% lower compared to our study (37.4 vs. 44.0 mm Hg), which could be due to the different volume of added dead space (350 vs. 1200 mL). In both their and our studies, this led to respiratory acidosis, as evidenced by lower blood pH and higher P_*ET*_CO_2_. It is possible that lowering pH and increasing pCO_2_ stimulate the sympathetic response to splenic contraction, releasing erythrocytes into the bloodstream, and thereby increasing the kinetics of oxygen uptake (Richardson et al., 2008). Furthermore, despite the lack of hematocrit measurement in our study, following the conclusions by Barbieri et al. (2020), we can assume that the governing mechanism is the Bohr effect. The increase in hematocrit and hemoglobin content in the blood (HHb), observed in their study, suggests that hemoglobin is used to buffer excess hydrogen ions, while Woorons et al. (2010) identified a key role for bicarbonates. Higher bicarbonate concentrations may be the source of adaptation, delaying the onset of metabolic acidosis and improving buffering capacity, as also observed in our study (Table 2). It may also be beneficial for pH regulation and development of the ability to produce energy through anaerobic metabolism (Woorons et al., 2010), allowing more lactate to accumulate in muscles (Chycki et al., 2018). This is particularly important, because the reduction in energy available from anaerobic glycolysis, the accumulation of H^+^ in muscles, and the increase in extracellular potassium are major regulators of fatigue during high-intensity exercise, such as maximal sprinting (Glaister et al., 2008). Thus, the use of warm-up under conditions of elevated pCO_2_ may provide a protective mechanism against the onset of premature fatigue and may even enhance exercise readiness. Several studies have found that hypercapnia can lead to lower lactate release into the blood (Ehrsam et al., 1982; Graham et al., 1986), and respiratory acidosis resulting from hypercapnia can shift (delay) the linear efflux of lactate from muscle. Yamamoto et al. (1988) reported a greater increase in blood lactate during restitution after hypoventilation induced by reduced-frequency breathing (RFB), compared to normal breathing. Woorons et al. (2007) had similar observations in their study, demonstrating delayed lactate efflux from active muscles during prolonged expiration (PE) exercise. Prolonged release of the lactate ions may alter the glycolysis state and induce higher concentrations in working muscles, without its concomitant presence in the blood. However, this requires confirmation at the biopsy level.

Increase in body temperature is one of the main effects of warming up.

As muscle temperature increases, VO_2_ kinetics (Poole and Jones, 2012) and muscle glycogen degradation rates increase (Fink et al., 1975). In our study, re-inhalation of expiratory air, located in the added respiratory dead space volume mask, likely underwent prior heating in the lungs and led to body temperature increase (Table 2). This is particularly important, as increased temperature affects performance and improves lower extremity muscle strength by up to 3% for every 1°C increase in temperature (Sargeant, 1987). Obtaining more than 1% higher PPO after warm-up with ARDSv compared to the control condition in our study, confirms the foregoing reports, as the temperature difference between conditions was ∼0.6°C. It seems reasonable to measure the temperature of the air inside the ARDSv in the future as well. Performing statistically significantly greater work, at a lower VO_2_mean (Figure 2 and Table 3), during SIE_*ARDSwu*_ reinforces the foregoing hypotheses. According to Parolin et al. (1999), energy requirements during short, acute exercise are met mainly by phosphocreatine hydrolysis. Moreover, the kinetics of PCr recovery has been shown to be sensitive to manipulations of oxygen availability (Haseler et al., 1999). Higher oxygen utilization during warm-up before SIE may induce increased energy stores (PCr—phosphocreatine), which may explain the higher PPO in the first bout (non-significant) (Figure 2). Significantly decreased mean oxygen uptake during the main part indicates presumably lower reliance on aerobic energy in the sprint interval exercise after warm-up with added respiratory dead space volume. On the other hand, it did not have a negative influence on the anaerobic performance and energy stores restoration, because total work performed is higher after SIE_*ARDSwu*_. Although the lower VO_2_ might have resulted in faster VO_2_ kinetics at the beginning of the subsequent exercise bout (DiMenna et al., 2008). Further research are needed to detailed understanding these results.

In our earlier study (Danek et al., 2020a), we also observed performing more work during the sprint interval exercise using ARDSv in the main part. In their study, Citherlet et al. (2021) used the Wim Hof breathing method, a combination of hyperventilation followed by voluntary breath-holding at low pulmonary volume, before repeated sprints (the RAST test). However, despite large physiological effects (reduction of blood saturation to approximately 60%, onset of respiratory alkalosis), a single WHBM session did not improve anaerobic performance in repeated sprint exercise (no change in peak and mean power and FI). Future studies could directly compare these methods and confront the mechanism of temperature rise and hypercapnic increase in bicarbonate concentration with respiratory alkalosis. As mentioned earlier, further studies should verify, e.g., the effects of ARDS_*V*_ breathing on the oxygenation and deoxygenation levels of working muscles, using near-infrared spectroscopy, as well as the effects on phosphocreatine resynthesis rate or removal of metabolites, such as inorganic phosphate, during SIE.

Our results indicate that breathing air with altered composition increases P_*ET*_CO_2_ and pCO_2_ in the blood, presumably through stimulation of peripheral chemoreceptors, and leads to increased respiratory minute volume. Obtaining higher VE by increasing VT confirms previous explanations of this phenomenon after ARDSv (Smolka et al., 2014; Danek et al., 2020a; Szczepan et al., 2020). Granados et al. (2016) demonstrated that 20 min of treadmill exercise at an intensity of 60%VO_2_max using a modified version of ARM reduces ventilation (VE) and respiratory rate (Rf), which can lead to increased blood CO_2_ levels. Although this variable was not directly assessed, the use of ARM increased dead space volume (by 240 mL), while the built-in three mask resistance caps impeded inspiratory and expiratory flow. This led to a decrease in VE and peripheral oxygen saturation, as well as increased perceived exertion, as well as higher scores on the Back Anxiety Inventory (BAI), in adolescents and adults. When using ARDSv in the training of swimmers, Szczepan et al. (2020) did not observe changes in respiratory rate and respiratory muscle strength, whereas in our previous study (Danek et al., 2020a) in which we applied ARDSv during SIE on a cycle-ergometer, there was a significant increase in VE by increasing VT. Nevertheless, there was no change in inspiratory (PImax) or expiratory (PEmax) muscle strength. Despite the change in respiratory rhythm, we observed no change in respiratory muscle strength in our study (Figure 4). This suggests that ARDSv breathing is not a limiting factor in respiratory effort, despite the altered ventilation pattern—especially since the mean ventilation did not exceed 50% of the maximal value. The 1200 mL, 2.5-cm-diameter mask used does not appear to be a tool intended for respiratory muscle training (Shei, 2020). Nevertheless, this problem should be the subject of separate studies on the long-term adaptation of ARDS_*V*_ use. Additionally, we found no differences in Rf or RPE after ARDS_*V*_ breathing. Similar results were presented by Jung et al. (2019), where RPE did not differ between normal respiration and respiration with expiratory muscle training (ETM) during continuous cycling (50 and 70% VO_2_max). Thus, a 10-min warm-up with ARDSv is not perceived to be more difficult, which is important for its use in implementing a regular training strategy. Unfortunately, we did not measure other psychological characteristics, such as feelings of pleasure. However, protocols perceived as more pleasant are more likely to be selected and performed by practitioners (Townsend et al., 2017).

Taking the above, the research unveiled a new approach to warm-up enhancing ergogenic effects without additional fatigue induced by respiratory acidosis under hypercapnia conditions. From an applied perspective, sport scientists and coaches should keep in mind that, application of the added respiratory dead space volume 1200 mL through 15-min, provokes ergogenic effects based on increasing body temperature, increasing bicarbonate ions and buffering reserve without respiratory muscle fatigue. Thus, it is a suitable method, given the aforementioned mechanisms on sprint interval exercise performance. It may be crucial in competition demanding maximal sprint performance like speed skating, swimming, track, and field or track cycling. Interestingly, further research may examine potential effects during re-warm-up, especially maintaining and/or increasing body temperature and increasing bicarbonate ions and buffering reserve between warm-up and competition phase.

Some limitations of this study should be considered, despite the interesting results we have presented. The first was a relatively small group of physically active young men studied, therefore the results obtained should be transferred to representatives of other groups with caution (females, youth, the overweight, the obese, the elderly, etc.). Further studies are needed to confirm the ergogenic effect of using ARDSv during warm-up e.g., in a group of trained athletes. We realize that further, detailed studies are needed to explain mechanisms other than those we have outlined in the hypotheses, such as blood flow tests, continuous monitoring of blood saturation, hemoglobin oxygenation, and deoxygenation at the skeletal muscle level, as well as catecholamine levels that may affect cellular metabolism. Finally, the results of our experiment can be related to the sprint interval exercise protocol. Having considered the foregoing, there is a need to determine if similar effects will occur when performing repeated sprint exercise or high intensity interval training.

## Conclusion

Finally, use of a warm-up with added respiratory dead space volume mask leads to hypercapnia, increased body temperature, blood buffering capacity, and increased work capacity during sprint interval exercise sessions. Increasing respiratory system work during the warm-up does not lead to respiratory muscle fatigue, and therefore does not undermine the ergogenic effects induced. We recommend using this form of warm-up before regular high-intensity SIE training, because it can lead to greater long-term adaptations.

## Data Availability Statement

The raw data supporting the conclusions of this article will be made available by the authors, without undue reservation.

## Ethics Statement

The studies involving human participants were reviewed and approved by the University Research Ethics Committee (1/2019). The patients/participants provided their written informed consent to participate in this study.

## Author Contributions

ND, KM, and MZ designed the study and analyzed the data. ND and KM conducted the research. ND composed the manuscript. All authors edited the manuscript and approved the final draft.

## Conflict of Interest

The authors declare that the research was conducted in the absence of any commercial or financial relationships that could be construed as a potential conflict of interest.

## Publisher’s Note

All claims expressed in this article are solely those of the authors and do not necessarily represent those of their affiliated organizations, or those of the publisher, the editors and the reviewers. Any product that may be evaluated in this article, or claim that may be made by its manufacturer, is not guaranteed or endorsed by the publisher.
